# Depression and associated factors among HIV-positive youths attending antiretroviral therapy clinics in Jimma town, southwest Ethiopia

**DOI:** 10.1371/journal.pone.0244879

**Published:** 2021-01-06

**Authors:** Derara Girma, Sahilu Assegid, Yenealem Gezahegn

**Affiliations:** 1 Public Health Department, College of Health Sciences, Salale University, Fiche, Ethiopia; 2 Epidemiology Department, Institute of Public Health, Health Sciences College, Jimma University, Jimma, Ethiopia; Ohio State University, UNITED STATES

## Abstract

**Background:**

Depression is recognized as a prominent health problem and a growing public health concern in HIV-positive youths. Despite this fact, in Ethiopia, there is a dearth of evidence on the prevalence of depression and its associated factors among HIV-positive youths.

**Methods:**

A facility-based cross-sectional study was conducted from March 16 to June 01, 2020, among 331 HIV-positive youths attending antiretroviral therapy clinics in Jimma town. A systematic random sampling technique was used to enroll study participants. Bivariable and multivariable logistic regression was done to identify factors associated with depression. Variables with a p-value ≤0.25 on the bivariable analysis were candidates for multivariable analysis. Adjusted odds ratios with the respective 95% CI were calculated and p-value <0.05 were used to set statistically significant variables in the multivariable analysis.

**Results:**

Out of a total of 331 sampled HIV positive youth, 325 have participated in this study with a response rate of 98.2%. The prevalence of depression was 30.2% (95%CI:25.2%-35.1%). Female sex (AOR = 4.12, 95%CI:2.28–7.47), history of hospital admission (AOR = 2.45, 95%CI:1.28–4.70), discontinued education due to HIV/AIDS illness (AOR = 2.09, 95%CI:1.12–3.90), poor treatment adherence (AOR = 2.23, 95%CI:1.04–4.78), opportunistic infections (AOR = 2.16, 95%CI:1.17–3.97), high baseline viral load (AOR = 3.35, 95%CI:1.82–6.16) and ≤6 months duration of HIV diagnosis (AOR = 3.14, 95%CI: 1.47–5.72) were factors significantly associated with depression.

**Conclusion:**

This study demonstrated a high prevalence of depression among HIV-positive youths. Factors such as female sex, treatment non-adherence, opportunistic infections, <six months since diagnosed with HIV, hospitalization history, high baseline viral load, and school discontinuation due to HIV/AIDS were significantly associated with depression. Therefore, we recommend regular screening for depression co-morbidity among HIV-positive youths and linkage with mental health service providers.

## Introduction

Depression is the common neuropsychiatric comorbidity of HIV/AIDS illness [1], and WHO predicts that both diseases are expected to be the leading causes of disease burden by 2030 [2]. Currently, in the circumstance of HIV/AIDS, depression is recognized as the global mental health challenge for HIV-positive youths [3]. Consistently, studies across low-middle and high-income countries among HIV-positive youths have documented high rates of depression ranging from 33.8–63% [4–8]. Despite this fact, depression is found under-diagnosed [9] and untreated among HIV positive patients [10]

Depression can lead to thoughts of death and suicide, with a confirmed 800,000 people death each year globally [11] and the second most common cause of death among youths [12]. Particularly, depression is established as a risk factor for ART non-adherence, low rates of viral suppression, and difficulties in treatment retention among HIV-positive youths [3]. Furthermore, the serious consequences of depression in HIV-positive youths also continue into adulthood; wherein it is associated with poor quality of life and increasing the risk of HIV/AIDS transmission to others [13], faster disease progression, and also earlier death [14].

Moreover, studies conducted among HIV-positive youths so far showed factors such as; rural residence [15], substance use (alcohol, cigarette) [8,16], non-adherence to ART [7,17], older age category in youth [7,18], being female [19], non-disclosure of serostatus [20], hospitalization history [21], HIV-related stigma [22], lack of social support, and presence of opportunistic infections [18] were found to heighten the risk of depression.

Despite those recognized entanglements, the lack of screening and information on how to intervene, prevent or improve mental health disorders including depression remains a major barrier for LMICs, wherein the bulk of HIV-positive youths live and the epicenter of HIV-related youths mortality in the world [23]. In Sub-Saharan Africa, depression in young people with HIV/AIDS is a major yet neglected public health problem prompting more studies to screen and treat to improve HIV outcomes [24]. The same is true in Ethiopia, the limited access to comprehensive HIV/AIDS mental health services remains a major challenge to efficiently combat depression in HIV-positive youths [25].

Youths are stated as a major vulnerable group to HIV/AIDS and also account for a large percentage of all HIV/AIDS cases in Ethiopia [26]. Living with chronic diseases, like HIV/AIDS may upsurge the likelihood of depression comorbidity. In Ethiopia, depression in HIV-positive youths has not been adequately investigated. However, the studies conducted in Addis Ababa revealed depression in 35.5% [18] and 31.7% [27] of HIV-positive youths. Even so, only urban dwellers and referral hospital attendants have been recruited for the studies, which lacks the generalizability of evidence in the country. In meantime, the socio-demographic and economic variation could differ depression levels in HIV/AIDS patients [28]. Moreover, further research in HIV-positive youths is suggested in previous studies [18,27].

Given a limited number of studies scrutinizing prevalence and factors associated with depression among HIV-positive youths in Ethiopia, assessing depression and associated factors would lead to early recognition, a decline of depression burden, and guidance in the development of an effective strategy.

## Methods

### Study design and setting

A facility-based cross-sectional study was carried out at public health facilities that provide HIV/AIDS care and treatment services in Jimma town from March 16 to June 01, 2020. These include Jimma Medical Center, Shenen Gibe General Hospital, Jimma Health Center, and Higher2 Health Center. All of the health facilities are providing regular HIV/AIDS care and treatment service possessing separated anti-retroviral therapy clinics. Jimma town is one of the town administrations of Oromia regional state and located at the distance of 356 km in the southwest direction of Addis Ababa, the capital city of Ethiopia. The total number of HIV-positive youths attending antiretroviral therapy clinics were 664.

### Participants

All HIV-positive youths (15–24 years old) who were attending antiretroviral therapy clinics in Jimma town were the source population. All HIV-positive youths (15–24 years old) who were attending antiretroviral therapy clinics in Jimma town during the study period and who had at least one previous visit at antiretroviral therapy clinics were eligible for the study.

### Sample size determination and sampling techniques

**Fig 1:**
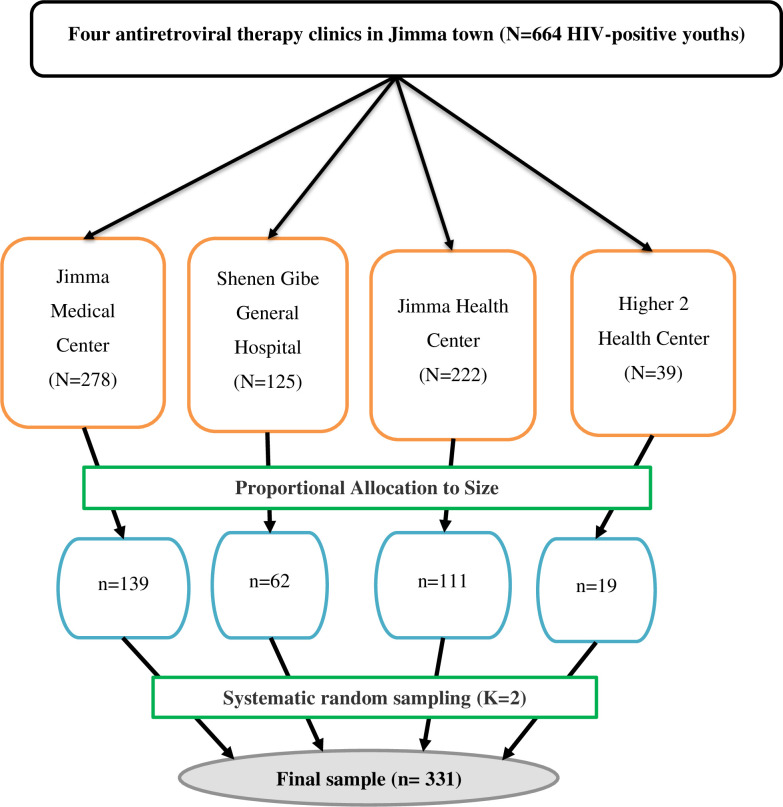


The sample size was calculated using the formula for estimation of a single population proportion in Epi Info STAT CALC. version 7.2 with the assumptions of 95% confidence level (CL), margin error (d) of 0.04, and 0.355 prevalence (P) of depression was taken from the previously conducted study [18]. Thus, after adding 10% of the non-response rate, the final sample size obtained was 331. Then, the sample size was distributed using proportional allocation to size (PAS) to each antiretroviral therapy clinics in the town. Finally, a systematic random sampling technique [K = N/n, yielding a sampling interval of two] was applied to recruit the study participants. The first patient to be interviewed was selected using the lottery method within the interval. Finally, the determined sample in each clinic was achieved through exit interviews with every K^th^ interval (Fig 1: Schematic diagram showing the sampling procedure).

### Data collection procedures

Data were collected using a pretested structured questionnaire via face-to-face interview to capture information on socio-demographic characteristics, stressors (past-traumatic) factors, behavioral factors, psycho-social and caregiver related factors, and clinical factors. Medical records of HIV-positive youths were reviewed to extract data on clinical and HIV-related factors. Data were collected by BSc nurses and onsite supervision was done by psychiatrists on daily basis within ten weeks of working days.

### Measurements

Depression was measured by a structured Patient Health Questionnaires-9 (PHQ-9). PHQ-9 is a nine-item tool that is directly based on the nine diagnostic criteria for major depressive disorder in the DSM-IV. The status of the study participant was determined as depressed if the PHQ-9 score is ≥10 [7,16]. PHQ-9 has acceptable reliability with a Cronbach’s-alpha of 0.89, a sensitivity of 88%, and specificity of 88% for depression diagnosis [29]. Furthermore, PHQ-9 is a reliable and validated tool in Ethiopian patients living with HIV/AIDS and showed good internal and test re-test reliability with Cronbach’s alpha of 0.85 and an intraclass correlation coefficient of 0.92 [30]. A cut-off score of PHQ-9 ≥10 can be used to diagnose depression regardless of age category (for sub-groups) with a sensitivity of 0.88 and specificity of 0.85 [31]. PHQ-9 tool was also reliable in this study (Cronbach-alpha = 0.81). The 3-item Oslo‐3 Social Support Scale (OSS‐3) was used to measure HIV positive youth family/social support [18]. The tool was reliable in this study (Cronbach-alpha = 0.79). Stigma was measured by the 8-item short version of the HIV stigma scale. The tool consisted of questions concerning disclosure status, negative self-image, and public attitudes [32]. The tool was reliable in this study (Cronbach-alpha = 0.87). Adherence was calculated based on the number of prescribed pills and missed pills in the past fourteen days before data collection day. Then, based on WHO guidelines to measure optimal treatment adherence, patients who had an intake of ≥95% of the prescribed medication were considered as good adherence, those who had an intake of <95% were classified as poor adherence [33]. The wealth index was measured by a simplified and updated Ethiopian wealth index equity tool using principal component analysis. The tool contains 15 simplified household assets questions available from www.equitytool.org. Accordingly, the wealth index of the 1^st^ and 2^nd^ quintiles were classified as poorest (40%), those in the 3^rd^ quintile were middle (20%), and those in the 4^th^ and 5^th^ quintiles were richest (40%) [34]. The tool was reliable in this study (Cronbach-alpha = 0.80). Physical activity was assessed using two questions of a brief physical activity assessment tool. Each question was independently measured and the summation of the two questions was used to determine the sufficiency level of physical activity. Accordingly, those patients who have scored ≥4 were categorized as sufficiently active otherwise insufficiently active [35]. The tool was reliable in this study (Cronbach-alpha = 0.71). Additionally, various socio-demographic characteristics, stressors (past-traumatic) factors, behavioral factors, psycho-social and caregiver related factors, and clinical factors were assessed as explanatory variables.

### Data quality control

PHQ-9 is a validated, translated to Afan Oromo, and culturally adapted tool [36]. Besides, all questionnaires were translated to the local languages, (Afan Oromo) by two independent bilingual translators and back-translated to English to guarantee consistency. The one-day training was given to the data collectors. The supervisors were also trained on how to monitor the data collection procedures. Ten percent (10%) of the questionnaire was pre-tested at Agaro general hospital and the relevant clarifications and corrections on vague points were made as required. Also, a reliability test was done and tools with Cronbach-alpha >0.7 were used in the actual data collection.

### Data processing and analysis

Data were entered into Epi Data version 3.1 and exported into SPSS version 25 for analysis. Data exploration was carried out to assess the completeness and descriptive statistics were used to describe the study participants’ data based on its nature. Bivariable analysis and crude odds ratio with a 95% confidence interval was used to realize the association between an independent variable and the outcome variable using binary logistic regression. All independent variables with a p-value ≤0.25 in the bivariable analysis were simultaneously included in the multivariable analysis using a backward stepwise method to identify factors independently associated with depression. The adjusted odds ratio with a 95% confidence interval was computed to measure the strength of the association. Finally, a p-value <0.05 was declared a statistically significant variable. The model fitness was checked using the Hosmer and Lemeshow goodness-of-fit model and the model was fitted (p = 0.307).

### Ethical consideration

Ethical clearance was obtained from the Institution Review Board of the institute of health of Jimma University. The permission letter was obtained from the administrative office of each antiretroviral therapy clinic. Informed written consent for youths ≥18 years old and assent for youths <18 years old was obtained from each study participant. Parents/caregivers' permission was obtained for youths <18 years old. Privacy and confidentiality were maintained.

## Results

### Socio-demographic characteristics

A total of 325 HIV-positive youths participated in this study with a response rate of 98.2%. More than half, 199 (61.2%) of the study participants were male. The mean age of the respondents was 20.23 with (2.8 SD). The majority, 252 (77.5%) were urban residents (Table 1).

**Table 1:** Sociodemographic characteristics of HIV positive-youths attending antiretroviral therapy clinics in Jimma town, southwest Ethiopia, 2020 (n = 325).

Variable	Category	Number (%)
**Sex**	Male	199 (61.2)
	Female	126 (38.8)
**Age**	15–19	124 (38.2)
	20–24	201 (61.8)
**Educational status**	No formal education	22 (6.8)
	Primary (1–8)	152 (46.8)
	Secondary (9–12)	108 (33.2)
	Diploma and above	43(13.2)
**Education discontinuation due to HIV/AIDS illness**	Yes	103(31.68)
	No	222(68.32)
**Residence location**	Urban	252 (77.5)
	Rural	73 (22.5)
**Occupation status**	Unemployed	67(20.6)
	Gov’t employee	33(10.2)
	Self-employed	70(21.5)
	Student	155(47.7)
**Household wealth index level**	Lowest class	127(39.1)
	Middle class	68(20.9)
	Higher class	130(40.0)
**Types of primary caregiver**	Both parents	107(48.0)
	Single parent	67(30.0)
	Others**	49(22.0)

**siblings, grandparents, relatives, orphanages.

### Clinical and psycho-social support characteristics

About, 51 (15.7%) of the participants had a duration of below six months since diagnosed with HIV/AIDS. Fifty-four (16.6%) had poor medication adherence and about two-thirds of the study participants, 216 (66.5%) were in the WHO non-advanced clinical stage (stage I and II). Almost half of the participants 162 (49.8%) had ≥1000 copies/ml baseline viral load. More than a third, 116 (35.7%) had an opportunistic infection. The commonest opportunistic infections were; Mycobacterium tuberculosis 30 (9.2%) followed by pneumonia 17(5.2%) and oral candidiasis 15 (4.6%). One hundred twenty-eight (39.4%) were stigmatized and far more than half, 192 (59.1%) had poor social support. One-fourth, 87 (26.8%) did not disclose their sero-status. Almost a third, 104 (32%) of the study participants had a history of hospital admission in the past twelve months (Table 2).

**Table 2:** Clinical and psychosocial characteristics of HIV-positive youths attending antiretroviral therapy clinics in Jimma town, southwest Ethiopia, 2020 (n = 325).

Variable	Category	Number (%)
**Treatment adherence**	Poor adherence	54(16.6)
	Good adherence	271(83.4)
**Opportunistic infection(s)**	Yes	116(35.7)
	No	209(64.3)
**Baseline viral load (copies/ml)**	≥1000	162(49.8)
	<1000	163(50.2)
**Current WHO clinical stage**	Stage-I	145(44.6)
	Stage-II	71(21.8)
	Stage-III	69(21.2)
	Stage-IV	40(12.3)
**Baseline CD4 count (cell/mm**^**3**^**)**	<200	25(7.7)
	≥200	300(92.3)
**History of adverse drug reaction**	Yes	42(12.9)
	No	283(87.1)
**Duration of HIV/AIDS**	≤6 months	51(15.7)
	>6 months	274(84.3)
**Stigmatized**	Yes	128(39.4)
	No	197(60.6)
**Social support**	Poor	192(59.1)
	Intermediate	89(27.4)
	Strong	44(13.5)
**Hospitalization history**	Yes	104(32.0)
	No	221(68.0)

### Prevalence of depression

The prevalence of depression among HIV-positive youths attending Jimma town ART clinics was 30.2% (95% CI:25.2%-35.1%). Of the 30.2% depression level; 18.8% were in moderate depression, 8.6% were in moderately severe depression, and 2.8% were in severe depression.

### Factors associated with depression among HIV positive-youths

In bivariable analysis; sociodemographic variables (sex, residence), stressors (history of hospital admission in the past twelve months, failed school term, death of biological parents, discontinued education due to HIV/AIDS illness, stigma), behavioral variables (smoking, khat chewing, alcohol drinking), and clinical variables (treatment adherence, baseline viral load, history of TB treatment, presence of opportunistic infections, Efavirenz based regimen, baseline CD4 count and duration since HIV diagnosis) were significantly associated with depression and candidate for multivariable analysis. In the multivariable model, the covariates: sex (AOR = 4.12, 95%CI: 2.28–7.47), history of hospital admission in the past twelve months (AOR = 2.45, 95%CI: 1.28–4.70), discontinued education due to HIV/AIDS illness (AOR = 2.09, 95%CI: 1.12–3.90), treatment adherence (AOR = 2.23, 95%CI: 1.04–4.78), opportunistic infections (AOR = 2.16, 95%CI: 1.17–3.97), baseline viral load (AOR = 3.35, 95%CI: 1.82–6.16), and duration of HIV diagnosis (AOR = 3.14, 95%CI: 1.47–5.72) were independently associated with depression among HIV-positive youths. (Table 3)

**Table 3:** Factors associated with depression among HIV-positive youths attending antiretroviral therapy clinics in Jimma town, southwest Ethiopia, 2020 (n = 325).

Variable	Category	Depression		COR (95% CI)	AOR (95% CI)
		Yes (%)	No (%)		
**Sex**	Male	38(19.1)	161(80.9)	1	1
	Female	60(47.6)	66(52.4)	3.85[2.34–6.33]	4.12[2.28–7.47]**
**Residence location**	Urban	71(28.2)	181(71.8)	1	1
	Rural	27(37.0)	46(63.0)	1.50[0.86–2.59]	1.06[0.49–2.27]
**Hospital admission**	Yes	47(45.2)	57(54.8)	2.75[1.67–4.52]	2.45[1.28–4.70]*
	No	51(23.1)	170(76.9)	1	1
**Failed school terms/classes**	Yes	30(35.7)	54(64.3)	1.54[0.90–2.64]	1.01[0.50–2.04]
	No	58(26.5)	161(73.5)	1	
**Death of biological parents**	Yes	42(41.2)	60(58.8)	2.09[1.27–3.43]	1.29[0.68–2.47]
	No	56(25.1)	167(74.9)	1	1
**Discontinued school due to HIV/AIDS illness**	Yes	39(40.6)	57(59.4)	2.21[1.31–3.71]	2.09[1.12–3.90]*
	No	49(23.7)	158(76.3)	1	1
**Alcohol drinker**	Yes	34(41.5)	48(58.5)	1.98[1.17–3.35]	1.07[0.46–2.47]
	No	64(26.3)	179(7)	1	1
**Cigarette smoker**	Yes	15(42.9)	20(57.1)	1.87[0.914–3.8]	0.66[0.24–1.83]
	No	83(28.6)	207(71.4)	1	1
**Khat chewer**	Yes	36(44.4)	45(55.6)	2.35[1.39–3.97]	1.33[0.67–2.64]
	No	62(25.4)	182(74.6)	1	1
**Physical activity level**	Insufficient	93(32.7)	191(67.3	3.51[1.33–9.23]	1.93[0.65–5.68]
	Sufficient	5(12.2)	36(87.8)	1	1
**Stigmatized**	Yes	45(35.2)	83(64.8)	1.47[0.91–2.38]	0.79[0.42–1.52]
	No	53(26.9)	144(73.1)	1	1
**Treatment adherence**	Poor	27(50.0)	27(50.0)	2.82[1.55–5.12]	2.23[1.04–4.78]*
	Good	71(26.2)	200(73.8)	1	1
**Baseline CD4 count (cell/mm**^**3**^**)**	<200	12(48.0)	13(52.0)	2.29[1.01–5.23]	1.36[0.45–4.09]
	≥200	86(28.7)	214(71.3)	1	1
**Baseline viral load (copies/ml)**	≥1000	66(40.7)	96(59.3)	2.81[1.71–4.63]	3.35[1.82–6.16]**
	0–1000	32(19.6)	131(80.4)	1	1
**Opportunistic infections**	Yes	52(44.8)	64(55.2)	2.88[1.76–4.70]	2.16[1.17–3.97]*
	No	46(22.0)	163(78.0)	1	1
**Duration of HIV/AIDS**	≤6 months	25(49.0)	26(51.0)	2.65[1.44–4.88]	3.14[1.47–5.72]*
	>6 months	73(26.6)	201(73.4)	1	1
**Efavirenz based regimen**	Yes	49(36.8)	84(63.2)	1.70[1.05–2.75]	1.19[0.64–2.23]
	No	49(25.5)	143(74.5)	1	1
**History of MTB treatment**	Yes	15(45.5)	18(54.5)	2.09[1.01–4.36]	1.12[0.39–3.38]
	No	83(28.4)	209(71.6)	1	1

** significant at p-value <0.001.

* significant at p-value <0.05. 1 = references.

## Discussion

This study assessed the prevalence of depression and its associated factors among HIV-positive youth. The study suggests that nearly one-third, (30.2%) of HIV-positive youths were suffering from depression. The finding from this study is in line with the previous studies conducted among HIV-positive youths in Thailand (27.8%) [37], Tanzania (27%) [15], South Africa (33.8%) [38], and Ethiopia (31.7%) [27]. However, this finding is lower than the previous studies done among HIV-positive youth in Uganda (46%) [33], Kenya (52.6%) [7], and Ethiopia (35.5%) [18]. The current integration of mental health services into existing HIV care might reduce depression [39].

Conversely, this finding is much higher than the study conducted among HIV-positive adults in Ethiopia such as, 20% in Dessie [40], 14.6% in Aksum [41], and 11.7% in Debre Markos [42]. Exposure to psychosocial stress, developmental, and hormonal factors will increase depression in youths but not in adults [43]. Additionally, this finding is higher than the study among HIV-positive youths in England (16%) [44], Ukraine (13%) [45], Thailand (15%) [16], Malawi (18.9%) [20], and Nigeria (20%) [21]. The difference may be attributable to the use of different psychometric scales, study settings, and also applying different exclusion criteria.

Female sex, history of hospital admission, discontinued education due to HIV/AIDS illness, treatment non-adherence, opportunistic infections, high baseline viral load, and duration of HIV diagnosis were significantly associated with depression. The greater chance of depression among females than males in our study was similar to the reports of other studies in the USA [19,46] and South Africa [47]. Variation in hormonal fluctuation, psychological effects, and socio-cultural events may heighten the risk of depression in females [48].

History of previous hospital admission had increased the risk of developing depression. Increased frequency and severity of complications, detaching from the usual environment and social support among hospitalized youths will result in psychological distress. This finding is similar to studies from Nigeria [21] and the USA [49]. HIV-positive youths who discontinued school due to HIV/AIDS illness were more likely to develop depression. Anxiety and anger due to underperforming in school give a strong sense of hopelessness. The previous studies conducted in Kenya [7] and Malawi [20] confirmed this.

Treatment non-adherence was significantly associated with depression. Treatment non-adherence would result in the emerging of drug-resistant strains of HIV, fastening disease progression, and increasing the incidence of mental health illnesses. It is supported by studies from Kenya [7] and Ethiopia [18]. The presence of opportunistic infection(s) had increased the risk of depression. Depression can result from dissatisfaction with one's physical appearance due to opportunistic infection(s) and the administration of different overlapping drugs in its treatment. This is consistent with the studies from Ethiopia [18], Rwanda [17], Tanzania [50], and the USA [51].

HIV-positive youths who had the baseline viral load of ≥1000 copies/ml were at high risk of depression. This result is supported by two studies from the USA [19,52]. The high viral load is a marker of poor disease progression and immune functioning resulting in depression [53]. Being less than six months since diagnosed with HIV is significantly associated with depression. This is supported by a study from the USA [54]. This is due to adjustment reaction to recent notification of seropositivity, admit reliance on health care, and supposed social stigma and discrimination.

## Conclusion

This study demonstrated a high prevalence of depression among HIV-positive youths according to the national youth health strategy target [55]. Factors such as female sex, treatment non-adherence, opportunistic infections, <six months since diagnosed with HIV, hospitalization history, high baseline viral load, and school discontinuation due to HIV/AIDS were significantly associated with depression. Therefore, we recommend regular screening for depression co-morbidity among HIV-positive youths and linkage with mental health service providers. Additionally, the scale-up of integrated mental health services working in conjunction with HIV/AIDS programs is required to avert depression among HIV-positive youth.

### Limitation of the study

The limitation of this study is social desirability bias. Because the data collection method was self-report, participants might have given socially acceptable responses, especially to substance use-related questions.

## Supporting information

S1 File English version questionnaires. (DOCX)

S1 Dataset Minimum data set. (XLS)

## Abbreviations

AOR: Adjusted Odds Ratio
CI: Confidence Interval
COR: Crude Odds Ratio
DSM-IV: Diagnostic Statistical Manual Fourth edition
LMICs: Lower and Middle-Income Countries
WHO: World Health Organization
