# Supplementary material for: Depression and associated factors among HIV-positive youths attending antiretroviral therapy clinics in Jimma town, southwest Ethiopia
Source: PLoS One. 2021 Jan 6;16(1):e0244879. doi: 10.1371/journal.pone.0244879 (PMC7787463; doi:10.1371/journal.pone.0244879)
Supplement: S1 File — (DOCX) [file pone.0244879.s001.docx]

## English version questionnaires

| **Part-I: Socio-demographic characteristics** | | | | | | | | | |
| --- | --- | --- | --- | --- | --- | --- | --- | --- | --- |
| S. No | Questions | | | Responses | | | Skip patterns | | |
| 101 | Respondent sex | | | 1. Male 2. Female | | |  | | |
| 102 | Age (In a completed year) | | | _____________ | | |  | | |
| 103 | Do you have a formal education? | | | 1. No 2. Yes | | | If “no” go-to Q105 | | |
| 104 | If “yes” to the Q103 (encircle only the highest grade completed) | | | 1. 1-4 grade 2. 5-8 grade 3. 9-12 grade 4. Diploma and above | | | See at Q304 &305 | | |
| 107 | Occupation | | | 1. Unemployed 2. Government employee 3. Self-employed 4. Student 5. Others (specify)_____________ | | |  | | |
| **Household wealth index questionnaires** | | | | | | | | | |
| S. No | Question | | | Options | | | Remarks | | |
|  | Residence location | | | 1. Urban 2. Rural | | |  | | |
| Q1 | Does your household have electricity? | | | 1. No 2. Yes | | |  | | |
| Q2 | Does your household have a radio? | | | 1. No 2. Yes | | |  | | |
| Q3 | Does your household have a television? | | | 1. No 2. Yes | | |  | | |
| Q4 | Does your household have a refrigerator? | | | 1. No 2. Yes | | |  | | |
| Q5 | Does your household have an electric *“mitad”*? | | | 1. No 2. Yes | | |  | | |
| Q6 | Does your household have a table? | | | 1. No 2. Yes | | |  | | |
| Q7 | Does your household have a chair? | | | 1. No 2. Yes | | |  | | |
| Q8 | Does your household have a bed with a cotton /sponge/ spring mattress? | | | 1. No 2. Yes | | |  | | |
| Q9 | Does any member of this household have a bank account? | | | 1. No 2. Yes | | |  | | |
| Q10 | What is the main source of drinking water for members of your household? | | | 1. Piped to yard/plot 2. Others | | |  | | |
| Q11 | What kind of toilet facility do members of your household usually use? | | | 1. Pit latrine without slab/open pit 2. No facility/bush/field 3. Others | | |  | | |
| Q12 | What type of fuel does your household mainly use for cooking? | | | 1. Wood 2. Electricity 3. Others | | |  | | |
| Q13 | What is the main material of the floor in your household? | | | 1. Earth/sand 2. Others | | |  | | |
| Q14 | What is the main material of the exterior walls in your household? | | | 1. Bamboo with mud 2. Others | | |  | | |
| Q15 | What is the main material of the roof in your household? | | | 1. Metal/corrugated iron 2. Others | | |  | | |
| **Part-II: Caregiver related factor** | | | | | | | | | |
| 201 | Do you have a primary caregiver in your home who looks after you? | | | 1. No 2. Yes | | | If “no” go-to Q301 | | |
| 202 | Type of a primary caregiver | | | 1. Both parents 2. Only a mother 3. Only a father 4. Siblings 5. Other (specify) _____________ | | |  | | |
| 203 | Do your primary caregiver changed since you diagnosed with HIV/AIDS? | | | 1. The caregiver has changed once or more 2. No change in a caregiver | | |  | | |
| **Part-III: Stressors/past-traumatic events factors** | | | | | | | | | |
| 301 | Did you have a history of hospital admission in the past 12 months? | | | 1. No 2. Yes | | |  | | |
| 302 | Did you disclose your HIV sero-status to others? | | | 1. No 2. Yes | | |  | | |
| 303 | Did you ever fail school term/class? | | | 1. No 2. Yes | | |  | | |
| 304 | Did you ever discontinue school due to HIV/AIDS illness? | | | 1. No 2. Yes | | |  | | |
| 305 | Did you experience the death of biological parents? | | | 1. No 2. Yes | | | If “no” go-to Q401 | | |
| 306 | If “yes” to Q305 who is passed way? | | | 1. Both (mother and father) 2. Mother only 3. Father only | | |  | | |
| **Part-IV: behavioral factors** | | | | | | | | | |
| S. No | Questions | | Response | | | | | | Skip patterns |
| In your life which of the following substances have you ever used (non-medical use only)? | | | | | | | | | |
| 401 | Alcohol | | 1. Yes 2. No | | | | | |  |
|  | Cigarette | | 1. No 2. Yes | | | | | |  |
|  | Khat | | 1. No 2. Yes | | | | | |  |
|  | Shisha (marijuana) | | 1. No 2. Yes | | | | | |  |
|  | Others (specify) | | ______________________ | | | | | |  |
| In the past 3 months, how often have you used the substances you mentioned above? | | | | | | | | | |
| 402 | Alcohol drinking | | 1. Never 2. Once or twice in the last 3 months 3. 1 to 3 times per month in the last 3 months 4. 1 to 4 times per week in the last 3months 5. 5 to 7 days per week in the last 3 months | | | | | |  |
|  | Cigarette smoking | | 1. Never 2. Once or twice in the last 3 months 3. 1 to 3 times per month in the last 3 months 4. 1 to 4 times per week in the last 3months 5. 5 to 7 days per week in the last 3 months | | | | | |  |
|  | Khat chewing | | 1. Never 2. Once or twice in the last 3 months 3. 1 to 3 times per month in the last 3 months 4. 1 to 4 times per week in the last 3months 5. 5 to 7 days per week in the last 3 months | | | | | |  |
|  | Shisha (marijuana) | | 1. Never 2. Once or twice in the last 3 months 3. 1 to 3 times per month in the last 3 months 4. 1 to 4 times per week in the last 3 months 5. 5 to 7 days per week in the last 3 months | | | | | |  |
| **Physical activity measuring questions** | | | | | | | | | |
| 403 | How many times a week, do you usually do 20 minutes of vigorous physical activity that makes you sweat or puff and pant? (for example, jogging, heavy lifting, digging, aerobics, or fast bicycling) | | 1. ≥3 times/week  2. 1–2 times/week  3. none | | | | | |  |
| 404 | How many times a week, do you usually do 30 minutes of moderate physical activity or walking that increases your heart rate or makes you breathe harder than normal? (for example, mowing the lawn, carrying light loads, bicycling at a regular pace, or playing ball) | | 1. ≥5 times/week 2. 3-4times/week 3. 1-2 times/week 4. None | | | | | |  |
| **Part-V: psychosocial support factors** | | | | | | | | | |
| The following questions are related to the availability of social support. Those questions provide whom you can count on for help or support. Describe the person’s relationship to you. | | | | | | | | | |
| S. No | | Questions | | | | Response | | | |
| 502 | | How many people are so close to you that you can count on them if you have serious problems? | | | | 1. None 2. 1 or 2 3. 3 to5 4. 6 or more | | | |
| 503 | | How much concern do people show in what you are doing? | | | | 1. No concern and interest 2. Little concern and interest 3. Uncertain 4. Some concern and interest 5. A lot of concern and interest | | | |
| 504 | | How easy can you get practical help from neighbors if you should need it? | | | | 1. Very difficult 2. Difficult 3. Possible 4. Easy 5. Very easy | | | |
| The following questions are related to stigma. All of the following statements refer to the way you feel (not what you think others think about you) since you were diagnosed with HIV   \| S. No \| **How Do You feel about yourself?** \| **Options** \| \| \| \| \| --- \| --- \| --- \| --- \| --- \| --- \| \| Strongly disagree \| Disagree \| Agree \| Strongly agree \| \| 505 \| Having HIV makes me feel unclean \| 1 \| 2 \| 3 \| 4 \| \| 506 \| Having HIV makes me feel I'm a bad person \| 1 \| 2 \| 3 \| 4 \| \| 507 \| Having HIV in my body feels disgusting \| 1 \| 2 \| 3 \| 4 \| \| 508 \| Most people think a person with HIV is disgusting \| 1 \| 2 \| 3 \| 4 \| \| 509 \| Most people with HIV are rejected when others learn \| 1 \| 2 \| 3 \| 4 \| \| 510 \| Most people believe a person who has HIV is dirty \| 1 \| 2 \| 3 \| 4 \| \| 511 \| I am very careful whom I tell that I have HIV \| 1 \| 2 \| 3 \| 4 \| \| 512 \| I work hard to keep my HIV a secret \| 1 \| 2 \| 3 \| 4 \| | | | | | | | | | |
| **Part-VI: Clinical factors questionnaires and checklist** | | | | | | | | | |
| S. No | | Questions | | | Response | | | Skip pattern | |
| 601 | | Have you ever missed ARV drug doses over the last 2 weeks? | | | 1. No 2. Yes | | | If no go to Q603 | |
| 602 | | If “yes” to Q601 how many numbers of ARV drug doses missed over the last 2 weeks? | | | ________ (in number) | | |  | |
| 603 | | Number of ARV drug doses had to be taken over the last 2 weeks | | | ________ (in number) | | |  | |
| 604 | | Baseline CD4 cell count | | | __________ (cell/µl) (from card) | | |  | |
| 605 | | Baseline viral load | | | ________________ (number of copies/mL) (from card) | | |  | |
| 606 | | Current WHO clinical stage | | | 1. Stage-I 2. Stage-II 3. Stage-III 4. Stage-IV (from card) | | |  | |
| 607 | | Presence of opportunistic infections | | | 1. No 2. Yes (from card) | | | If no go to Q609 | |
| 608 | | If “yes” to Q607 specify it | | | ___________________ (from card) | | |  | |
| 609 | | Current ART regimen | | | 1. 1^st^ line regimen 2. 2^nd^ line regimen 3. 3^rd^ line regimen (from card) | | |  | |
| 610 | | efavirenz-based regimen | | | 1. No 2. Yes (from card) | | |  | |
| 611 | | History of ARV side effects | | | 1. No 2. Yes | | |  | |
| 612 | | History of TB treatment | | | 1. No 2. Yes | | |  | |
| 613 | | Duration since HIV diagnosis | | | _______________ (in a completed months) (from card) | | |  | |

**Part-VII: Questionnaire for depression measuring (PHQ-9)**

**Instruction:** this interview consists of nine items. Please listen carefully to what I am going to read each item of statement for you, and then tell me the one number in each item that best describes the way you have been feeling during the **past two weeks**.

| **S. No** | **Questions** | **Respondents possible answers** | | | |
| --- | --- | --- | --- | --- | --- |
|  |  | **Not at all** | **Several days** | **More than half the days** | **Nearly every day** |
| 701 | Little interest or pleasure in doing things | 0 | 1 | 2 | 3 |
| 702 | Feeling down, depressed, or hopeless | 0 | 1 | 2 | 3 |
| 703 | Trouble falling or staying asleep, or sleeping too much | 0 | 1 | 2 | 3 |
| 704 | Feeling tired or having little energy | 0 | 1 | 2 | 3 |
| 705 | Poor appetite or overeating | 0 | 1 | 2 | 3 |
| 706 | Feeling bad about yourself or that you are a failure or have let yourself or your family down | 0 | 1 | 2 | 3 |
| 707 | Trouble concentrating on things, such as reading the newspaper or watching Television | 0 | 1 | 2 | 3 |
| 708 | Moving or speaking so slowly that other people could have noticed? Or the opposite being so fidgety or restless that you have been moving around a lot more than usual | 0 | 1 | 2 | 3 |
| 709 | Thoughts that you would be better off dead or of hurting yourself in some way | 0 | 1 | 2 | 3 |
| PHQ-9 difficulty measure | | | | | |
| If you are experiencing any of the problems in this form, how difficult have these problems made it for you to do your work, take care of things at home or get along with other people?  1. Not difficult at all 2. Somewhat difficult 3. Very difficult 4. Extremely difficult | | | | | |

THANK YOU!

I have finished my interview.
